# Iron Transporters and Ferroptosis in Malignant Brain Tumors

**DOI:** 10.3389/fonc.2022.861834

**Published:** 2022-04-21

**Authors:** Jingyu Zhao, Yaqi Wang, Lei Tao, Ligong Chen

**Affiliations:** ^1^ School of Pharmaceutical Sciences, Key Laboratory of Bioorganic Phosphorus Chemistry and Chemical Biology (Ministry of Education), Tsinghua University, Beijing, China; ^2^ Collaborative Innovation Center for Biotherapy, State Key Laboratory of Biotherapy and Cancer Center, West China Hospital, West China Medical School, Sichuan University, Chengdu, China; ^3^ Advanced Innovation Center for Human Brain Protection, Beijing Tiantan Hospital, Capital Medical University, Beijing, China

**Keywords:** iron transport, transporters, ferroptosis, malignant brain tumors, therapeutic strategy

## Abstract

Malignant brain tumors represent approximately 1.5% of all malignant tumors. The survival rate among patients is relatively low and the mortality rate of pediatric brain tumors ranks first among all childhood malignant tumors. At present malignant brain tumors remain incurable. Although some tumors can be treated with surgery and chemotherapy, new treatment strategies are urgent owing to the poor clinical prognosis. Iron is an essential trace element in many biological processes of the human body. Iron transporters play a crucial role in iron absorption and transport. Ferroptosis, an iron-dependent form of nonapoptotic cell death, is characterized by the accumulation of lipid peroxidation products and lethal reactive oxygen species (ROS) derived from iron metabolism. Recently, compelling evidence has shown that inducing ferroptosis of tumor cells is a potential therapeutic strategy. In this review, we will briefly describe the significant regulatory factors of ferroptosis, iron, its absorption and transport under physiological conditions, especially the function of iron transporters. Then we will summarize the relevant mechanisms of ferroptosis and its role in malignant brain tumors, wherein the role of transporters is not to be ignored. Finally, we will introduce the current research progress in the treatment of malignant brain tumors by inducing ferroptosis in order to explain the current biological principles of potential treatment targets and treatment strategies for malignant brain tumors.

## 1 Introduction

Brain tumors can be categorized as primary malignant types and secondary forms from metastasis (1). Of these, roughly 40% will be malignant and the incidence rate of malignant brain tumors is higher in males (2, 3). Primary brain tumors are the first common tumor and the first cause of tumor death in children (3). Brain tumors can be classified based on origin, such as glioblastoma (GBM), neuroblastoma and meningioma (4). GBM is the most common and aggressive malignant primary brain tumor, with a limited response to the current standard of treatment. Most GBM patients can only live up to 15-20 months (5).

Malignant brain tumors are commonly intratumoral heterogenic, which likely explains their poor clinical prognosis of malignant brain tumors poor and easy to relapse (6). Despite current multimodality treatment efforts, combining in surgical resection when feasible, with radiotherapy, chemotherapy and symptomatic treatment, the median survival remains short (7).

Iron is necessary for life (8). Iron plays an extremely significant role in brain development and function, and is involve in many biological processes such as embryonic neuronal development, myelin formation, neurotransmitter synthesis and oxidative phosphorylation (9, 10). Iron deficiency impairs the function of iron-requiring enzymes in all tissues, however, excessive iron accumulation leads to toxicity through oxidative stress activation of cell death signaling pathways (11). To maintain adequate and safe amounts of iron levels, cells express a the coordination of a wide variety of proteins, which tightly control both intracellular and systemic iron metabolism (12). Iron transporters participate in the regulation of iron uptake, storage and distribution, wherein help maintain iron homeostasis (13).

Ferroptosis is an iron-dependent form of regulated cell death (14). The intracellular iron homeostasis and balance between the oxidation and reduction of phospholipids is tightly associated with ferroptosis. Ferroptosis occurs when iron overload induces lipid peroxidation (11). Recent studies showed that ferroptosis is involved in the death of pathological cells in malignant brain tumors, which may have a therapeutic potential towards malignant brain tumors (15, 16). The specific way of ferroptosis inhibiting cancer may be to induce oxidative stress and resist treatment antagonism of cancer cells, in which iron transporters may has a stronger role. Although great progress has been made in the study of the biological function and disease correlation of ferroptosis, its biological signal pathway and underlying mechanism remain to be elucidated.

Starting from the iron transport in the body under physiological conditions, we further summarize the specific mechanism of iron metabolism disorder and ferroptosis in the pathological condition of malignant brain tumors, in particular, the crucial role of transporters. Finally, we summarized the specific mechanisms and targets for inducing ferroptosis in the treatment of malignant brain tumors and introduced potentially related drugs.

## 2 Iron Physiology

### 2.1 Iron and Iron Transporters

#### 2.1.1 Iron Function

Iron is a vital micronutrient for nearly all living organisms due to its significant role in many biological processes such as catalyzing redox reactions and transporting oxygen. In addition, iron is essential for the functions of many enzymes and prosthetic groups (17, 18).

#### 2.1.2 Dietary Sources of Iron

Iron is required across all human life stages, from embryological development, to infancy or old age. Estimated daily average iron requirements are the highest in pregnancy 3rd trimester (19). Despite having an efficient iron recycling mechanism, humans need to absorb about 10% of our total iron needs from regular dietary to maintain normal health. Dietary iron exists as either heme iron or non-heme iron. Heme iron is derived from hemoglobin, myoglobin and neuroglobin found in animal foods, and its absorption is not affected by diet; meanwhile non-heme iron is found mainly in plant foods, and its absorption is influenced by inhibitors and enhancers found in the diet. Nonetheless both are affected by iron storage levels in the body (19, 20).

#### 2.1.3 Iron Absorption

The absorption site of iron is mainly in the mucosa of duodenum and upper jejunum. In a nutshell, iron absorption can be divided into two steps; first iron in food enters intestinal mucosal cells, second iron in intestinal mucosal cells crosses the cell membrane into capillaries and is transported systemically to the whole body in bloodstream (19).

#### 2.1.4 Iron Transport

In humans, a number of proteins have evolved which tightly regulate iron homeostasis since we cannot rapidly excrete iron in the urine and iron must be transported and stored intracellular on a protein carrier due to extremely low free iron levels both systemically and intracellular (21). These includes the proteins that are involved in iron transport, both in the circulation and intracellularly, the reductases and oxidases that facilitate the movement of iron across cell membranes, and other proteins that regulate these processes (22). Iron transporters are vital role to maintain iron homeostasis in the body, and a total of 22 iron transporters have been identified (**Table 1**). The functions of several iron transporters are introduced below.

**Table 1:** Iron Transporters. For detailed information about the gene tables, please visit: http://www.bioparadigms and http://www.org.genecards.org.

Human gene name	Protein name	Aliases	Substrates	Tissue and cellular expression	Sequence accession ID	Mouse KO model
SLC11A1	NRAMP1	NRAMP1 NRAMP LSH	Mn^2+^, Fe^2+^, other divalent metal ions	Phagolysosomes of phagocytes (macrophages, neutrophils)	NM_000578.4	No
SLC11A2	DMT1	NRAMP2 DCT1	Fe^2+^, Cd^2+^, Co^2+^, Cu^1+^, Mn^2+^, Ni^2+^, Pb^2+^, Zn^2+^	Widespread, including intestine (duodenum), erythroid cells, kidney, lung, brain, testis (Sertoli cells), thymus	NM_001174125.2	Yes
SLC22A17	BOIT	BOCT, NGALR	1-methyl-4-phenyl-pyridinium (MPP (+)), Fe	Brain	NM_020372.4	No
SLC25A28	Mitoferrin 2 (Mfrn2)	MRS3/4, MRS4L	Fe^2+^	Ubiquitous (heart, liver, kidney)	NM_031212.4	No
SLC25A37	Mitoferrin 1 (Mfrn1)	HT015, MSC, MSCP	Fe^2+^	Fetal liver, bone marrow, spleen, placenta, liver, brain	NM_016612.4	No
SLC39A14	ZIP14, LZT-Hs4	ZIP14 KIAA0062 NET34	Zn, Fe, Mn, Cd	Widespread, liver	NM_001128431.4	Yes
SLC40A1	MTP1, IREG1	Ferroportin 1(FPN1)	Fe^2+^	Duodenum, macrophages, liver Kupffer cells, placenta, kidney	NM_014585.6	Yes
SLC41A1	MgtE	MgtE, NPHPL2	Mg^2+^ (Sr^2+^, Zn^2+^, Cu^2+^, Fe^2+^, Co^2+^, Ba^2+^, Cd^2+^)	Kidney, heart, testis, skeletal muscle, prostate, adrenal gland, thyroid	NM_173854.6	Yes
SLC41A2	SLC41A1-L1, SLC41A1-like 1	SLC41A1-L1	Mg^2+^ (Ba^2+^, Ni^2+^, Co^2+^, Fe^2+^, Mn^2+^)	Highest expression in cerebellum, lymph nodes, stomach, lungs, testis, skin	NM_032148.6	No
SLC46A1	PCFT	HCP1	Reduced folates, folic acid, antifolates, heme	Small intestine, choroid plexus, kidney (proximal tubule), liver (sinusoidal), placenta	NM_080669.6	No
SLC48A1	HRG-1	HHRG-1 HRG1, HRG-1	Heme	Liver, heart, CNS, kidney, skeletal muscle, small intestine	NM_017842.3	No
SLC49A1	FLVCR1	FLVCR, MFSD7B, AXPC1, PCARP	Heme	Ubiquitous, high expression in intestine, liver, kidney, brain, bone marrow	NM_014053.4	No
SLC49A2	FLVCR2	MFSD7C, CCT, EPV, PVHH, FLVCRL14q	Heme	Liver, kidney, brain, lung, placenta, fetal liver, bone marrow	NM_017791.3	No
SLC57A1	NIPA1	NIPA1, SPG6, FSP3	Mg^2+^, Sr^2+^, Fe^2+^, Co^2+^	Constitutively express at low levels, significant enrichment in the brain (human); widely expressed, including heart, kidney, liver, colon, less in the brain, not in the small intestine (mouse)	NM_144599.5	No
SLC57A3	NIPAL1	NIPA3	Mg^2+^, Sr^2+^, Ba^2+^, Fe^2+^, Cu^2+^	Biased expression in esophagus, skin and 13 other tissues	NM_207330.3	No
SLC58A2	TUSC3	N33	Mg^2+^, Fe^2+^, Cu^2+^, Mn^2+^	Placenta, pancreas, testis, ovary, heart, prostate	NM_006765.4	No
TF	TF	Transferrin HEL-S-71p, PRO1557, PRO2086, TFQTL1	Fe^2+^	Liver	NM_001063.4	No
ABCB6	ABCB6	ABC, LAN, MTABC3, PRP, umat	Iron	Ubiquitous expression in testis, ovary and 25 other tissues	NM_005689.4	No
ABCB7	ABCB7	ABC7, ASAT, Atm1p, EST140535	Iron	Ubiquitous expression in duodenum, heart and 25 other tissues	NM_004299.6	No
ABCB8	ABCB8	MITOSUR M-ABC1 MABC1 EST328128	Organic and inorganic molecules	Mitochondria, cardiac	NM_001282291.2	No
ABCG2	ABCG2	BCRP ABCP MXR EST157481 CD338	Protoporphyrin IX (PPIX), heme, sphingosine-1-P	Biased expression in small intestine, duodenum and 12 other tissues	NM_004827.3	Yes

Transferrin (TF) is regulator of free iron levels in body fluids, binding, sequestering, and transporting Fe^3+^ ions. This iron carrier protein helps maintain iron availability systemically and prevents tissue oxidative damage caused by excessive free radical accumulation (23).

SLC25A37 (Mitoferrin 1, Mfrn1) is a solute carrier localized in the mitochondrial inner membrane. When iron enters cells, Mfrn1 transport iron into mitochondria, which is used to synthesize mitochondrial heme and iron sulfur clusters. Mitoferrin-1 is necessary for neuronal energy metabolism and influences brain function (24).

SLC11A2 (Divalent metal cation transporter 1, DMT1), is a proton-dependent iron importer of Fe^2+^, is involved in systemic iron recycling and cellular iron absorption. DMT1 is located on the parietal membrane of duodenal intestinal epithelial cells, where it brings dietary free iron into cells and promotes iron absorption (25). DMT1 is also involved in transferrin/transferrin receptor 1 (TF/TFR1) pathway, wherein transports iron absorbed by this pathway from the endosome into the cytosol (26).

SLC40A1 (Ferroportin 1, Fpn1), is a major iron export protein, is expressed in many cells, such as placental syncytiotrophoblasts, wherein plays a role in transferring maternal iron to the fetus and releasing iron from tissue into the blood. It should be noted that inactivating the murine Fpn1 gene globally is embryonic lethal (27).

### 2.2 Brain Iron Transport

#### 2.2.1 Brain Iron Function

Iron in the brain plays a crucial role in maintaining normal physiological function through its participation in many cellular activities such as mitochondrial respiration, myelin synthesis, neurotransmitter synthesis and metabolism (10). Iron is also essential in enzymes involved in the production of monoamines (dopamine, epinephrine, norepinephrine and serotonin), which are involved in social emotional development, executive function and memory processes. Therefore, maintaining iron homeostasis is essential for normal physiological activity of the brain (28).

Blood-brain barrier (BBB) and blood cerebrospinal fluid barrier (BCSFB) are of great significance to maintain the relative stability of physical and chemical factors in the internal environment of brain tissue and prevent harmful substances in blood from entering brain tissue (29). The BBB and BCSFB also controls iron transport from the bloodstream to the brain parenchyma, allowing for some independence of brain iron levels from the total body iron and providing some resistance to systemic iron toxicity (30, 31). Different cells types in the brain acquire iron through different pathways, which involving a myriad iron transporters (**Table 2**) (29).

**Table 2:** Proteins Involved in Brain Iron Transport.

Gene name	Fe species bound	Presence in					Function
		BBBBCSFB	Neurons	Microglia	Astrocytes	Oligodend-rocytes	
TF (Transferrin)	Fe^3+^	+	+	+	+	+	Transport iron to cells
DMT1 (SLC11A2)	Fe^2+^	+	+	+	+	+	Involved in iron absorption
Zip14 (SLC39A14)	Fe^2+^				+		Transporter of NTBI
FPN1 (SLC40A1)	Fe^2+^	+	+	+	+	+	Iron export from cells
CP (Ceruloplasmin)	Fe^2+^	+	+		+		Peroxidation of Fe^2+^ to Fe^3+^
HEPH (Hephaestin)	Fe^2+^	+	+	+		+	Peroxidation of Fe^2+^ to Fe^3+^
Ferr (Ferritin)	Fe^3+^	+	+	+		+	Intracellular iron storage protein

"+" refers to the existence of corresponding genes.

Herein we provide a summary of recent literature unveiling the mechanism of iron transport and regulation across the BBB and BCSFB, as well as the characteristics of iron transport and metabolism in different cell types of the central nervous system (CNS) such as neurons, microglia, astrocytes, and oligodendrocytes.

#### 2.2.2 Iron and Iron Transporters in BBB and BCSFB

CNS is tightly sealed from the changeable milieu of blood by the BBB and the BCSFB (31). BBB is an heterogenous multicellular complex system. This system includes tightly connected endothelial cells and a unique basement membrane. In addition to the parenchymal basement membrane, the basement membrane also contains an ensheathment of astrocytic end-feet, pericytes and perivascular antigen-presenting cells (32). BCSFB lies at the choroid plexuses in the lateral, third and fourth ventricles of the brain where the choroid plexus epithelial cells of the nonporous capillary wall contain a special carrier system for transporting various substances. This system is responsible for the exchange of substances between cerebrospinal fluid (CSF) and blood, and transport across BBB and BCSFB is important for the entry of iron into brain (33, 34).

TF/TFR1 pathway may be the main route of iron transporter across the luminal (apical) membrane of the BBB. Additionally, non-transferrin-bound iron (NTBI) uptake from the blood through luminal DMT1 and H-ferritin uptake may be partly responsible for iron transport across the BBB. Iron transport across the abluminal (basal) membrane is a Fpn1/hephaestin (Fpn1/Heph) and/or Fpn1/ceruloplasmin (CP)-mediated process (35, 36).

TF/TFR1/DMT1 pathway is an important pathway for iron transport across the BCSFB. Furthermore, iron export from the choroid epithelium to the CSF is mediated by the Fpn1/CP or Fpn1/Heph pathways. Beyond restriction of the access of substances from the blood to the CSF, it is possible that the BCSFB has a bigger impact on iron removal from the brain than iron uptake into the brain (35–37).

#### 2.2.3 Iron and Iron Transporters in Neurons

Iron is essential for neuron development and function (38). First iron is an essential cofactor for enzymes involved in energy metabolism and amino acid biosynthesis. Iron also plays a significant role for division of embryonic neurons as it is a cofactor for the enzyme ribonucleotide reductase. In addition, during early embryonic development, the dysfunction of yolk sac cells caused by excessive iron uptake leads to the necrotic degeneration of neuroectodermal cells (39, 40).

The neuronal expression levels of the TFR1 reflects their need for iron (41). DMT1 is also expressed in neurons, suggesting that after transferrin binding, iron is transported to the cytoplasm through DMT1 (42). DMT1 is involved in hippocampal neuronal iron uptake during development and memory formation (43). The presence of NTBI in brain extracellular fluids suggests that neurons can also take up iron as transferrin-free iron (44). Fpn1 and Heph are involved in the output of iron from the neuron (45, 46).

#### 2.2.4 Iron and Iron Transporters in Microglia

Microglia have vital roles in brain development and CNS homeostasis, including programmed cell death, clearance of apoptotic newborn neurons, as well as pruning developing axons and synapses (47, 48). Microglia are immune cells of the CNS, which are implicated in brain inflammation and can modulate the transport and metabolism of essential metal iron according to the anti-inflammatory and pro-inflammatory environment (49).

The mechanism of iron transport in microglia has been addressed in cell culture. The different sources of cells include primary adult mouse microglia (49), primary 2-day-old Sprague-Dawley microglia, primary newborn Wistar rat microglia (50), primary C57BL/6 mice microglial (51) and BV-2 microglial cells (52). Microglial cells interact with both TF bound-iron (TBI) and NTBI. TBI is taken up *via* the TFR1/DMT1 pathway, and after the release of iron in the acidic milieu of the endosome, this is translocated into the cytosol by DMT1 or other transporters (53). For NTBI uptake, an endogenous cell surface ferrireductase reduces Fe^3+^ to Fe^2+^ for uptake by DMT1 in a pH-dependent manner at the cell surface (54).

#### 2.2.5 Iron and Iron Transporters in Astrocytes

Astrocytes are the most abundant glial cells in the brain (55). In healthy CNS tissue, astrocytes maintain homeostasis of extracellular fluids, provide energy substrates to neurons, modulate local blood flow, and play essential roles in synapse development and plasticity (56). In addition, astrocytic end-feet form intimate contacts with the abluminal side of brain capillary endothelial cells (BCECs) in all brain regions. This close relationship makes it denotes an important role in nutrient capture from the circulating blood such as iron (57). Astrocytes theoretically can transport iron directly from BCECs to neurons and oligodendrocytes through intracellular transport (58).

The TF cycle is probably not the main process by which astrocytes obtain iron from endothelial cells (59). It is more likely that DMT1 mediates some of this uptake, since this transporter is strongly expressed in the astrocyte end-feet contacting with BCECs directly. This suggests that astrocytes can potentially uptake NTBI directly from BCECs (57).

In addition, the zinc transporter Zip14 and resident transient receptor potential channels have been suggested to be involved in the uptake of NTBI by astrocytes (60). Fpn1 and CP are highly expressed on astrocytic cell membranes, and both proteins may be essential in iron mobilization from these cells into the extracellular brain space (61, 62).

#### 2.2.6 Iron and Iron Transporters in Oligodendrocytes

Oligodendrocytes create myelin sheaths for CNS axons, assist in the jumping and efficient transmission of bioelectric signals, maintain and protect the normal function of neurons (63, 64). Oligodendrocytes are the cells with the highest iron levels in the brain. Oligodendroglia cells require iron as a cofactor for several enzymes involved in the proliferation and differentiation of oligodendrocyte precursor cells (OPCs), as well as enzymes required for the production of cholesterol and phospholipids, which are essential myelin components (65, 66).

In oligodendrocytes, TF/TFR1/DMT1 pathway plays a significant role in iron transport in immature oligodendrocytes, however the proportion of iron transported by this pathway may decrease with the beginning of myelination (36). DMT1 is essential for OPC maturation and normal myelination in mouse brain, which is considered to be a crucial pathway for many cells to uptake NTBI (67). Extensive literature suggests that H-ferritin is the main source of iron in oligodendrocytes, conferring high buffering capacity for iron (68). Heph is expressed by mature oligodendrocytes and plays a role in iron efflux from these cells, but white and gray matter oligodendrocytes can regulate iron efflux differently; while white matter oligodendrocytes upregulate the expression of Cp in the absence of Heph, likely as a fail-safe mechanism, gray matter oligodendrocytes lacks such compensatory pathway (69).

## 3 Ferroptosis and Transporters in Malignant Brain Tumors

### 3.1 The Transport Mechanisms in Ferroptosis

Ferroptosis is a form of iron-dependent regulatory cell death distinguished from necrosis, apoptosis and autophagy (70), which can be triggered by the small-molecule compound erastin and RSL3 (71, 72). Iron and polyunsaturated fatty acids (PUFAs) act as raw materials for lipid peroxidation to promote the occurrence of ferroptosis (73, 74). While glutathione peroxidase 4 (GPX4) using glutathione (GSH) as the substrate effectively removes excess ROS through antioxidant mechanism and inhibits ferroptosis (75). The increase of intracellular iron content, the accumulation of ROS and excessive lipid peroxidation are crucial to induce ferroptosis (76). Ferroptosis is closely related to iron metabolism, amino acid metabolism and lipid metabolism in cells. Therefore, iron transporters and amino acid transporters involved in metabolism have a marked effect on the cell sensitivity to ferroptosis (70, 77).

#### 3.1.1 Iron Transporters in Ferroptosis

DMT1 and TfR1 are involved in the absorption of intracellular iron (78, 79), while Fpn1 transports iron from the cell to the blood (27). They are both ubiquitous and crucial proteins that regulate the iron content in cells and are essential for the maintenance of iron homeostasis (**Table 3**). Iron is essential for cell growth, but it can promote the formation of toxic ROS during ferroptosis. In the case of excessive iron in cells, Fe^2+^ and H_2_O_2_ can generate hydroxyl radicals (OH^-^) through Fenton reaction, promoting the oxidation of PUFAs on the cell membrane, greatly accelerating lipid peroxidation and ultimately causing cell damage or death (80). Therefore, increasing the expression of TFR1 or decreasing the expression of Fpn1 will increase the accumulation of iron in the cell and result in ferroptosis. DMT1 located on the lysosomal membrane mediates iron transfer and the inhibitors of DMT1 can kill cells by accelerating lysosomal iron overload and an increase of ROS production (81).

**Table 3:** The characteristics of ferroptosis-related transport protein associated with malignant brain tumors.

Gene symbol	Alias	Protein name	Subcellular	Substrates	Related Brain Cancer
SLC7A11	xCT	Cystine/glutamate transporter	Plasma membrane	Cystine, Glutamate	Glioblastoma, Neuroblastoma
SLC3A2	4F2hc	4F2 cell-surface antigen heavy chain	Lysosome, Plasma membrane	L-type amino	Glioblastoma, Neuroblastoma
SLC1A5	ASCT2	Neutral amino acid transporter B (0)	Plasma membrane	Glutamine	Glioblastoma
SLC38A1	SNAT1	Sodium-coupled neutral amino acid transporter 1	Plasma membrane	Glutamine	Glioblastoma
SLC11A2	DMT1	Natural resistance associated macrophage protein 2	Plasma membrane, Endosome, Mitochondrion	Fe^2+^	Glioblastoma
SLC40A1	Fpn1	Solute carrier family 40-member 1	Plasma membrane	Fe^2+^	Glioblastoma, Neuroblastoma
SLC39A14	ZIP14	Metal cation symporter ZIP14	Plasma membrane	Mn^2+^, Fe^2+^, Zn^2+^	
TFR1	TFRC	Transferrin receptor protein 1	Plasma membrane	Fe^3+^	Glioblastoma, Neuroblastoma

Recently identified ferroptosis-related iron transporters ZIP14 (SLC39A14) can transport manganese, iron and zinc (**Table 3**). However, its main function is to transport manganese ions, while iron ions are not the main transport substrate of ZIP14 under normal physiological conditions (82, 83). Only in the state of iron overload, ZIP14 exhibits the function of transporting iron ions and mediating ferroptosis (84).

#### 3.1.2 Amino Acid Transporters in Ferroptosis

The amino acid transporter system Xc^−^ on the cell membrane is composed of two core components, SLC7A11 (Solute Carrier Family 7 Member 11, xCT) and SLC3A2 (Solute Carrier Family 3 Member 2, 4F2hc), involved in the exchange of extracellular cystine (Cys2) by transporting intracellular glutamate (Glu) (**Table 3**) (70). Intracellularly, Cys2 will be reduced to cysteine (Cys), thereby promoting the synthesis of GSH, the cofactor of GPX4. As a central regulatory protein for ferroptosis, GPX4 can convert GSH to oxidized glutathione (GSSG) whilst also reducing lipid hydroperoxides (L-OOH) to lipid alcohols (L-OH), which is the main mechanism to prevent lipid peroxidation and inhibit ferroptosis (85). In fact, knockout and inactivation of GPX4 both contribute to ferroptosis (86). Ferroptosis inducer erastin can result in GSH depletion and GPX4 inactivation by inhibiting system Xc^−^ transport of cystine (71), while RSL3 directly induces ferroptosis by inhibiting the activity of GPX4 (72). Cys is the crucial limiting amino acid for intracellular GSH synthesis and GSH depletion directly affects the function of GPX4. Therefore, system Xc^−^ that participates in the uptake of Cys2 is considered to be one of the most critical regulators of ferroptosis. Recent studies suggest that regulation of TP53 (87), Nrf2 (15), ATF4 (88), BECN1 (89) or interferon γ(IFNγ) released by CD8^+^ T cells (90) significantly inhibits the system Xc^−^, leading to a decrease in GSH synthesis and ferroptosis.

The transmembrane transport of glutamine (Gln) is dependent on SLC1A5 (Solute Carrier Family 1 Member 5) and SLC38A1 (Solute Carrier Family 38 Member 1) (**Table 3**). After entering the cell, Gln is catalyzed by glutaminase (GLS) and broken down into Glu and ammonia in the mitochondria (91). Subsequently, Glu can be converted to α-ketoglutarate (α-KG) that is involved in the oxidative energy supply as an important intermediate for the tricarboxylic acid (TCA) cycle (92). Glu is an indispensable molecule for generating GSH, which can effectively scavenge intracellular ROS. In cancer cells, inhibition of ferroptosis has been shown to be associated with high levels of Gln (93). Although glutaminolysis promotes cancer cell growth, this metabolic process can also induce ferroptosis toward cell death (94). The pivotal role of dihydrolipoamide dehydrogenase (DLD) in prompting ferroptosis induced by cystine deprivation or cystine import inhibition has been recently confirmed. Apart from stimulating DLD to produce hydrogen peroxides, α-KG can be further converted into acetyl-CoA, facilitating fatty acid synthesis and lipid peroxidation-dependent ferroptosis (95). MIR137 (microRNA137) has also been recently identified as a negative regulator of erastin or RSL3-induced ferroptosis through down-regulation of SLC1A5 in melanoma cells (96).

### 3.2 Ferroptosis and Malignant Brain Tumors

In 2021, the World Health Organization (WHO) released the fifth edition of the Classification of Tumors of the Central Nervous System (CNS) (WHO CNS5). Among various brain tumors, childhood brain tumors, adult gliomas and meningiomas are currently the most common brain neoplasia. Neuroglioma is one of the common primary central nervous system tumors that originate from glial cells. GBM is the most malignant and deadliest type of neuroglioma (97). Neuroblastoma is the most common extracranial tumor in children and nearly half of neuroblastoma occurs in infants and young children under 2 years of age (98). Meningiomas are tumors originating from arachnoid cap cells, most of which are benign. However, about 3% meningiomas are malignant, including invasive meningiomas (99). The current treatment methods for malignant brain tumors mainly include surgical resection, radiotherapy and chemotherapy.

Recently increasing numbers of studies have shown that ferroptosis is associated with the pathological process of a variety of neurological diseases, including neurodegenerative diseases, neurotrauma and brain tumors (100). Nevertheless, there has been less research on brain tumors compared to the other types of tumors so far. It is undeniable that ferroptosis, a new form of non-apoptotic cell death, will open up new therapeutic avenues for eliminating brain tumor cells (101).

Soon after ferroptosis was defined, researchers injected iron-containing water into the rats transplanted with glioma-35 cells and then focused on treating the tumor site with radiotherapy (102). They found that the tumor volume in the experimental group was significantly smaller than that in the control group. Mechanistically, in a separate report, it is suggested that iron-containing water treatment before radiation induces glioma cell death through the combination of apoptosis and ferroptosis (103). Furthermore, ferroptosis is proved to be involved in the GBM cell death which can be induced by neutrophils. It appears that this process requires activation signals given by the tumor microenvironment. When mature neutrophils infiltrating into the tumors are activated, they will trigger lipid peroxidation by transferring myeloperoxidase into GBM cells and increase cellular ROS, finally causing tumor cell ferroptosis (104).

Although most ferroptosis-related studies have concentrated on gliomas, neuroblastoma, another malignant brain tumor, is gradually coming into focus. Research suggests that overexpression of Mitochondrial ferritin (FtMt) in dopaminergic neuroblastoma cell line SH-SY5Y cells can significantly inhibit erastin-induced ferroptosis (105). This is mainly due to FtMt-mediated inhibition of cellular labile iron pool (LIP) and the accumulation of cytoplasmic ROS which protects against effects of ferroptosis. In another study with SH-SY5Y, the ferroptosis inhibitor Ferrostatin-1 (Fer-1) was found to have a neuroprotective effect under Rotenone-induced oxidative stress conditions (106).

**Figure 1:**
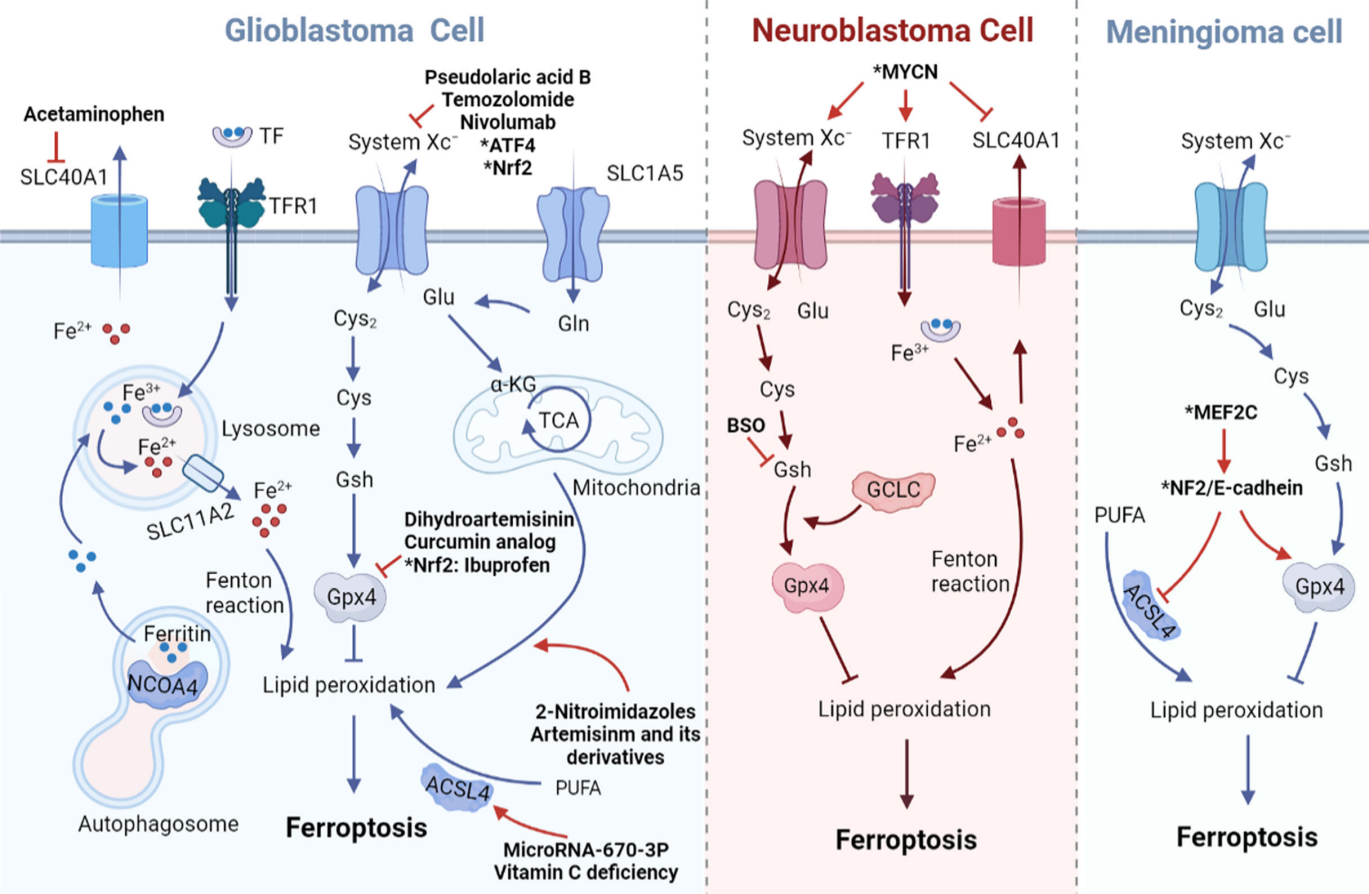
Impacts of ferroptosis-related transport proteins in three malignant brain tumor cells. In GBM cell, iron transport-related proteins DMT1 (SLC11A2), Fpn1 (SLC40A1), TFR1 and amino acid transporters system Xc^–^ (SLC7A11/SLC3A2), ASCT2 (SLC1A5) regulate the occurrence of ferroptosis all together. In MYCN-amplified neuroblastoma cell, lipid peroxidation and cell death are promoted due to increased expression of TFR1 and System Xc^–^ and lower expression of Fpn1. In meningioma cell, MEF2C mediated upregulation of NF2 and E-cadherin inhibits Erastin-induced ferroptosis. Arrows indicate promotion and blunt-ended lines indicate inhibition. Cys, cysteine; Cys2, cystine; GSH, glutathione; GPX4, glutathione peroxidase 4; Glu, glutamate; Gln, glutamine; TF, transferrin; TFR1, transferrin receptor 1; PUFA, polyunsaturated fatty acid; ACSL4, acyl-CoA synthetase long-chain family member 4; TCA, tricarboxylic acid cycle; α-KG, α-ketoglutarate; NCOA4, nuclear receptor coactivator 4; ATF4,activating transcription factor 4; Nrf2, nuclear factor erythroid-2-related factor; MYCN, BHLH Transcription Factor; MEF2C, Myocyte Enhancer Factor 2C; NF2, neurofibromatosis type 2; BSO, buthionine sulphoximine.

In a recently published study, researchers evaluated the expression of Merlin/Neurofibromin2 (NF2) and the ferroptosis regulator GPX4 in patients with primary meningioma and found a positive correlation between them. They speculated that the inactivation of NF2 in meningiomas may be more likely to cause ferroptosis. Furthermore, it has been determined that inhibition of NF2 and E-Cadherin can promote ferroptosis-related cytotoxicity and lipid peroxidation in meningioma cell lines. The transcription factor MEF2C has been shown to regulate the transcription of NF2 and E-cadherin genes. Silencing MEF2C, the expression levels of NF2 and E-cadherin in meningiomas decreased, which inhibited the growth of meningiomas mediated by ferroptosis (**Figure 1**). Therefore, MEF2C can be used as a potential molecular target for the treatment of aggressive meningiomas through modulating ferroptosis (107).

### 3.3 The Role of Transporters Associated With Ferroptosis in Malignant Brain Tumors

**Figure 2:**
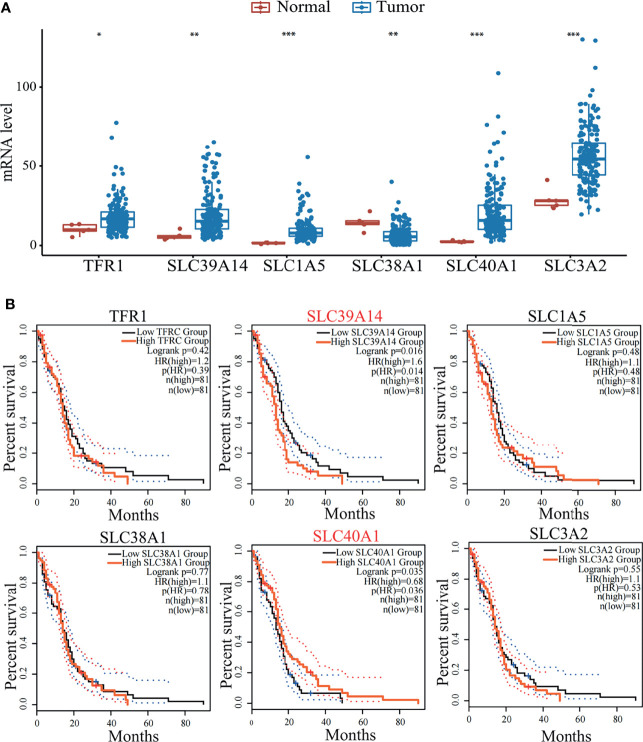
**(A)** Expression level of transporters (TFR1, SLC39A14, SLC1A5, SLC38A1, SLC40A1, SLC3A2) in tumor patients and normal people. Data mined from TCGA (https://cancergenome.nih.gov/). *p < 0.05, **p < 0.01, ***p < 0.001, compared with tumor patient group. **(B)** Survival curves of GBM patients mined from GEPIA2 (http://gepia2.cancer-pku.cn/). GBM patients were stratified into high or low expression groups based on the expression level of transporters (TFR1, SLC39A14, SLC1A5, SLC38A1, SLC40A1, SLC3A2) of patients. p<0.05 in Log‐rank test. OS, overall survival in months.

Ferroptosis plays a key role in the development of malignant brain tumors. As an important part of ferroptosis, relevant transporters can regulate amino acid metabolism and iron metabolism and are essential for the maintenance of iron homeostasis. Disorders of iron homeostasis in the brain will increase the risk of tumors, which may be one of the factors leading to the increased incidence of brain tumors (108). In addition, a group of ferroptosis-related genes have been discovered that may predict the prognosis of glioma patients based on clinical databases (109). In terms of iron metabolism, CDGSH iron-sulfur domain-containing protein 1 (CISD1) (110), poly r(C) binding protein 1 (PCBP1) (111) and transferrin (TF) (94) have a marked impact on ferroptosis by regulating the cellular content of iron. Here we compared the survival curve of brain tumor patients with the expression of ferroptosis-related genes and the results showed that the decrease in survival rate was related to the high-level expression of the protein required for iron intake (**Figure 2**). These data indicate that a better understanding of the role of ferroptosis-related transporters in malignant brain tumors may help provide more options for the treatment and prevention of brain tumors.

The obvious increase of lipid and cytoplasmic ROS is an important feature of ferroptosis and part of its regulatory factors have been used as small molecule drug targets to induce the death of cancer cells. Fpn1 can inhibit ferroptosis by reducing the accumulation of iron-dependent lipid ROS. Studies have found that in neuroblastoma cells, erastin induces the accumulation of iron and the low expression of Fpn1 involved in iron outflow (112). Furthermore, hepcidin, an amino acid peptide hormone (113) that binds with Fpn1 and stimulates Fpn1 degradation, increases antitumor activity of Erastin. This suggests that Fpn1 can be used as a potential therapeutic target for neuroblastoma in the future and Fpn1 inhibitors may provide a new approach for the treatment of neuroblastoma.

In neuroblastoma, gene amplification of the oncogenic transcription factor MYCN makes tumor cells more malignant and difficult to eliminate. Increased TFR1 expression and decreased Fpn1 expression in MYCN-amplified neuroblastoma cells results in high intracellular iron content. Overexpression of MYCN activates Xc^−/^GPX4 pathway, resulting in increased intracellular cystine and enhanced antioxidant protection (114). Therefore, the use of system Xc^−^ selective inhibitors or TFR1 agonists to treat MYCN-amplified neuroblastoma will increase the level of lipid peroxidation and eventually lead to ferroptosis of tumor cells (**Figure 1**).

In addition to neuroblastoma, GSH depletion caused by system Xc^−^ inhibition is associated with other malignant brain tumors (115). Nuclear factor (erythoid-derived)-like 2(Nrf2) overexpression or Kelch-like ECH associated protein 1(Keap1) knockdown can accelerate the growth of glioblastoma and promote the development of glioma cells (15). Similarly, xCT is positively regulated by Nrf2 and plays a crucial role in the inhibition of ROS accumulation during the ferroptosis process of glioma cells. Drug inhibitors targeting system Xc^−^ can rescue ROS generation, thereby increasing the sensitivity of glioma cells to ferroptosis and achieving the goal of treating malignant gliomas (**Figure 1**) (15).

The first-line treatment anti-tumor drug Temozolomide can inhibit the growth of glioblastoma. In order to explore the role of ferroptosis in this process, researchers treated human glioblastoma cell line TG905 cells with siRNA and found that knockdown of DMT1 reduced the level of ROS and iron production induced by Temozolomide (116). In addition, down-regulation of DMT1 also increased the expression of GPX4, Nrf2 and HO-1, thereby preventing the occurrence of ferroptosis. Temozolomide induces ferroptosis of some glioblastoma cells by increasing the expression of DMT1, so the divalent metal transporter DMT1 can be used as a drug target in glioblastoma.

## 4 Therapeutic Strategy

Mounting evidence suggests ferroptosis plays a beneficial role in tumors treatment. With the need for new treatments for malignant brain tumors, increased attention has been paid to drugs inducing ferroptosis that designed based on the regulatory pathways of ferroptosis. The main types of malignant brain tumors targeted by the novel Ferroptosis-based include GBM (117), fibrosarcoma (118), head and neck carcinoma (119).

Ferroptosis can be induced by increasing intracellular iron or ROS level (11). Inhibition of the glutathione peroxidase GPx4 or glutamate/cystine antiporter system Xc^−^ through the drugs is beneficial, promoting ferroptosis though increased ROS accumulation. Nrf2-Keap1 pathway promotes cell proliferation and diminishes ferroptosis (15). Although some studies have reported that inhibiting ferroptosis by activating Nrf2 pathway can play a neuroprotective role, for example, astrocytes protect neurons from ferroptosis by activating the Nrf2 pathway to supply neurons with GSTM2 and other antioxidants, inhibiting Nrf2 pathway in tumor cells to promote ferroptosis plays a therapeutic effect (120). ATF4 and Pseudolaric acid B promotes ferroptosis in a xCT-dependent manner (89, 121). Dihydroartemisinin initiates ferroptosis through GPx4 inhibition (122). Ibuprofen induces ferroptosis *via* downregulation of Nrf2-Keap1 signaling pathway (123).

Other mechanisms of promoting ferroptosis have also been reported, including activating the transcription factor BACH1 (BTB domain and CNC homology 1) (124) or Nox4 (121) to promote oxidative stress, inhibition of autophagy (125), vitamin C deficiency to reduce proliferation (126) and targeting ACSL4 which suppresses proliferation (127). Based on these mechanisms, related drugs have been found, such as 2-Nitroimidazoles, temozolomide, artemisinin and its derivatives.

Ferroptosis inducers may expand our arsenal of frontline therapeutic agents for combinatory approaches. Temozolomide toxicity operates is boost by ferroptosis (128). Androgen receptor (AR) ubiquitination is induced by the curcumin analog which suppresses growth of temozolomide-resistant GBM through disruption of GPX4-mediated redox homeostasis (129). Furthermore, T cell-promoted tumor ferroptosis is an anti-tumor mechanism, and targeting this pathway in combination with immunotherapy is another potential therapeutic approach (91, 130, 131). Nivolumab therapy revealed that clinical benefits correlate with reduced expression of SLC3A2 and increased IFNγ and CD8 (91).

Although many anticancer compounds that promote ferroptosis have been found, there are still many treasures to be discovered. Drugs targeting other mechanisms of ferroptosis need to be explored, such as targeted iron accumulation. A systematic assessment of the relationship between ferroptosis related genes (FRGs) expression profiles and the occurrence and development of tumors based on the Cancer Genome Atlas (TCGA), Chinese Glioma Genome Atlas (CGGA) datasets and FerrDb datasets may unveil new targets (77, 132). In fact, the potential impact of Acetaminophen in ferroptosis through interaction with CD44, HSPB1, and SLC40A1 was found this way (132).

To find out the potential correlation of GBM with transporters involved in ferroptosis. Here we compared the expression of several ferroptosis related transporters (SLC7A11, SLC3A2, SLC1A5, SLC38A1, SLC11A2, SLC40A1, SLC39A14, TFR1, TF) in normal people and GBM patients based on TCGA data. It is worth mentioning that the differentially expressed genes (DEGs) covered the majority of the transporters that we screened related to ferroptosis. Box-plot shows the expressions of SLC3A2, SLC1A5, SLC40A1, SLC39A14 and TFR1 increased significantly, the expression of SLC38A1 decreased significantly (**Figure 2A**). The effect of DEGs on the survival curve of GBM patients was further explored based on TCGA data (**Figure 2B**). As shown in the Kaplan‐Meier survival curve, median survival of GBM patients changed significantly according to the expression of SLC39A14 (p = 0.016) and SLC40A1 (p = 0.035), but the mechanism behind it remains to be explored. The above analysis further proves the potential of targeting these transporters and ferroptosis in the treatment of GBM.

Ferroptosis induction may prove as an effective therapeutic strategy against malignant brain tumors, yet a wide range of ferroptosis inducers are prone to off-target effects and may cause significant damage to normal cells. Therefore, it is urgent to develop tumor targeting delivery strategies of ferroptosis inducers. At present, many research are focusing on this aspect. Class I histone deacetylase (HDAC) inhibitors can selectively inhibit ferroptosis in neurons, but promote ferroptosis in tumor cells, which may be due to its different epigenetic regulation on the two cells. The combination of HDAC inhibitors and ferroptosis inducers can not only reduce the dosage of ferroptosis inducers to reduce toxicity, but also protect neurons (133, 134). Nano-targeting of WA allows systemic application and suppressed tumor growth due to an enhanced accumulation at the tumor site (135, 136).

At present, the treatment strategy targeting ferroptosis has been widely studied in various tumors, among which the advanced treatment strategy can potentially use for malignant brain tumors as well. Some new therapeutic mechanisms are worth learning. For example, gene interference by transferring genes with adeno-associated virus and iron nanoparticles enhance ferroptosis and inhibit tumor growth (137); ferroptosis inducer erastin or rsl3 is used independently or in combination with standard-of-care second-generation for the treatment of advanced prostate cancer (138); and activating ferroptosis by sequestering iron in lysosomes kills cancer stem cells (139). Studies have showed that targeted ferroptosis can used to overcome drug resistance of tumors. For example, Vorinostat promotes ferroptosis to overcome the resistance to epidermal growth factor receptor-tyrosine kinase inhibitors (EGFR-TKIs) (140); and artesunate inhibits growth of therapy-resistant renal cell carcinoma through induction of ferroptosis (141). Some advanced strategies are for targeted therapy. For example, Photodynamic therapy site-specifically produces reactive oxygen species for the Fenton reaction, which promotes ferroptosis and suppresses tumors (142); and catalytic nanomedicine that contains natural glucose oxidase and ultrasmall Fe_3_O_4_ nanoparticles selectively and effectively strengthens ferroptosis of tumor cells (143). In short, the essence can be drawn from the treatment of other tumors and used in the treatment of malignant brain tumors.

Inducing ferroptosis of tumor cells is a newly discovered strategy for the treatment of malignant brain tumors, but many problems remain to be solved, including elucidating the mechanism of ferroptosis in different malignant brain tumors, discovering new therapeutic targets for inducing ferroptosis of tumor cells, and increasing the tumor cell targeting of ferroptosis inducers. It is worth noting that the regulation of iron transport in tumor cells and the expression of transporters related to ferroptosis may have good therapeutic potential. Many transporters have become drug targets in recent years (144, 145). At the same time, clarifying iron transport under physiological conditions also provides an important research basis for targeted therapy of tumor cells, crucial to avoid the damage of normal tissues through off target effects.

## Author Contributions

LC proposed the research. JZ and YW both reviewed the literature and collected references. JZ, YW, and LT wrote the manuscript and finalized the paper. All authors contributed to the article and approved the submitted manuscript.

## Funding

This work was supported by National Natural Science Foundation of China (32130048, 92157301, 31971085 and 91857108 to LC), the Ministry of Science and Technology of China National Key R&D Programs (2018YFA0506903 to LC), Nation Science and Technology Major Projects for Major New Drugs Innovation and Development (2018ZX09711003-004-002 to LC), Tsinghua University Spring Breeze Fund (2021Z99CFY012 to LC), Tsinghua-Foshan Innovation Special Fund (2020THFS0133 to LC).

## Conflict of Interest

The authors declare that the research was conducted in the absence of any commercial or financial relationships that could be construed as a potential conflict of interest.

## Publisher’s Note

All claims expressed in this article are solely those of the authors and do not necessarily represent those of their affiliated organizations, or those of the publisher, the editors and the reviewers. Any product that may be evaluated in this article, or claim that may be made by its manufacturer, is not guaranteed or endorsed by the publisher.
